# Medical Expert and Medical Evidence

**Published:** 1875-04

**Authors:** M. A. McClelland

**Affiliations:** Knoxville, Ill.


A MONTHLY RECORD OF

*Medicine, Surgery and the Collateral Sciences.*

EDITED BY J. ADAMS ALLEN, M.D., LL.D.: AND WALTER HAY, M.D.

---

VOL. XXXII. — APRIL, 1875. — No. 4.

---

### Original Communications.

ARTICLE I.

### THE MEDICAL EXPERT AND MEDICAL EVIDENCE.

BY M. A. McCLELLAND, M.D., KNOXVILLE, ILL.

(Concluded from page 171.)

### WILLS.

Quite frequently the physician is called upon to write a will, and a discussion of the principles upon which these documents are based, will not be out of place in this connection.

The first great duty the physician must observe, is to ascertain the bodily and mental condition of the testator. "Less mind," it has been decided, (*Nichols* v. *Nichols*, 31 Vt. 331), "is ordinarily requisite to make a will, than a contract for sale," for the reason, "that in contracts of sale there are usually two parties, and some degree of antagonism between their interests and efforts;" while in making a will "one is left free to act upon his own perceptions merely."

"An understanding of the business about which the testator was engaged, of the kind and value of the property devised, and of the persons who were the natural objects of his bounty, and of the manner in which he wished to dispose of his property, are all the evidence, it has been ruled in Illinois, of the possession of testamentary capacity, unless the testator was affected with some morbid or insane delusion as to some one of those natural objects of his bounty." (W. & S. Med. Jurisp., §§ 19, 31.) If this unsoundness arises "from want of intelligence occasioned by defective organization, or by supervening physical infirmity, or the decay of advancing age, as distinguished from mental derangement," it alike incapacitates.

Mental soundness being presumed, the person writing the will is to limit himself to such inquiries as may enable him to understand the testator's wishes. These wishes he is to write down in the fewest, simplest and clearest words. The document is to have the name of the *place* where executed, with *exact* date of the transaction, and at the foot of the writing the signature of the testator, if possible, in his own handwriting.

The foregoing indications of "mental capacity" are given to guide the physician in respect to the propriety of drafting the will. When the document comes to be contested in court, the physician can be required to testify as to all the facts in the case, and he may give his opinion on "mental capacity" from facts observed by himself, but a physician not personally acquainted with the facts, must give his testimony on a hypothetical case, and is to express no opinion till he also knows the contents of the "will" in question. Even if the physician has observed the facts himself, unless he can state the grounds of his opinions, they are no better than surmises. Unless he is well informed upon these matters, he had better restrict himself to the functions of an ordinary witness, refusing to pass upon the question in issue on the ground of want of special study. By this course he will reflect honor upon himself and will assist in confirming popular confidence in the decisions of the law.

UNNECESSARY VISITS.

"Physicians are frequently charged with making unnecessary visits, and their bills are often paid very grudgingly, indeed, they may be able to collect them only by law. The investigation of such a case as this is a very difficult one, and the physician is to remember when called upon to testify in such a case, that the physician in attendance is the one properly to judge in these matters, each case being a law unto itself, and the responsibilities are his. The files of prescriptions, proving positive treatment of the disease said to have been diagnosed, would go far to prove the visits were necessary."

APHORISMS.

Any injury to the health or any death proved to have occurred subsequent to medical treatment, is to be imputed to the physician, when his treatment has been quite different from what is taught orally and in books, by his acknowledged scientific compeers, as the proper treatment, according to the rules of art, for such a case or one similar to it, and which is universally acknowledged by the medical experience of his compeers to be correct. (Caspar.)

"Life," in legal parlance, is equivalent to "respiration,"—not to have breathed is not to have lived. Therefore, to the question whether in a given case the fœtus *in utero* did not live, it should be replied that only life dependent upon *respiration* can be proved. (Caspar.)

"It is the right and duty of the forensic physician to give his testimony not only in accordance with the letter but also with the spirit of the law."

"In questions of legal responsibility, let the judge put special questions to the medical witness, referring to the individual case." (Bocker.)

"In respect to conditions upon which insanity depends, in by far the larger number of cases, *post mortem* examinations reveal some material changes. And if that material change is not noted, it should not be confidently asserted that no such change has taken place, but rather ascribe it to the inability of the observer, or to the want of those accessories of investigation that are necessary."

"A forensic physician, in a psychological case, has or may have two distinct functions, viz.: First, to determine how far a case of mental alienation is dependent upon bodily disease; and, second, to ascertain whether mental alienation is really present. To those who believe that there may be mental without physical disease, this proposition will be objectionable."

"Medical skill is not, in many instances, and without reference to the particular circumstances of the case, decisive as to the cause of death; and persons of science must, in order to form their own conclusions and opinions, rely partly on external circumstances." (Roscoe's Crim. Ev.)

*Experts.*—There is no established form for questions to experts. * * Any question may be proper which will elicit their opinions as to matters of science or skill, which are in controversy, and at the same time exclude their opinion as to the effect of the evidence in establishing controverted facts. (*Hunt* v. *Gaslight Co.*, 8 Allen.)

It is not admissible to permit a medical witness to give an opinion in a case where he has no means of ascertaining the facts in respect to which his opinion is asked. (*Millard* v. *Brown*, 35 N. Y.)

The opinion of an expert must be predicated on facts proved or admitted, or such as appear in evidence hypothetically stated. One not an expert must give facts, and circumstances within his own knowledge as the ground of his opinion. (*Roach* v. *Zehring*, 59 Pa.)

"The rules prescribing the qualifications of experts are established by law; but whether a witness offered as an expert has the legal qualifications which entitle him to testify in that capacity, is a question of fact to be decided by the court, in the exercise of its discretion, at the trial of the cause; and the decision of the presiding judge, in such a case, is not subject to exception or revision." (*Dole* v. *Johnson*, 50 N. H.; *Taylor* v. *Roger Williams Ins. Co.*, 50 or 51 N. H.)

"A physician, as a witness, cannot be allowed to express his professional opinion as necessarily to imply his belief of material facts outside of his art or profession, and not within his own knowledge, and which are not in their nature subject matter of mere opinion." (*Moore* v. *State of Ohio*, 17 O. S. R. 521; *McCurry* v. *Hooper*, 12 Ala. 823.)

"A physician cannot give an opinion as to whether another physician had acted honorably and faithfully; nor can he give an opinion on the opinion of another expert." (*Campbell* v. *Richards*, 5 B. & Adolph. 840; *Ramadge* v. *Ryan*, 9 Bingh. 333; *Heath* v. *Wing*, 45 Maine, 392; *Weatherbee's Executors* v. *Weatherbee's Heirs*, 38 Vt. 454.)

"A medical witness may, when the issue is sanity or insanity, be asked whether such and such symptoms proved by other witnesses, are, in his judgment, *symptoms* of insanity; but he cannot be asked if the act with which the defendant is charged is an insane act, for this is a fact to be decided by the jury." (*Rex* v. *Wright*, R. & R. 456; *Rex* v. *Searle*, 1 M. & Bab. 75; *Collet* v. *Collet*, 1 Curtis, 687; *Malton* v. *Nesbit*, 1 C. & P. 72.)

"It is not proper to inquire of a medical witness whether the person in question possessed sufficient capacity to transact business or to make a will." (*White* v. *Bailey*, 10 Mich. 155.)

"Special knowledge must be fully established before a witness can be examined as an expert." (*Sinclair* v. *Roush*, 14 Ind. 450; *Winans* v. *N. Y. & E. R. R. Co.*, 21 Howard U. S. 88; *Com.* v. *Wilson*, 1 Gray, 337; *Daniels* v. *Mosher*, 2 Mich. 183; *Lester* v. *Pittsfield*, 7 Vt. 161; *Mendum* v. *Com.*, 6 Rand. 704; *Elfelt* v. *Smith* 1 Minn. 125.)

"Experts may be asked as to hypothetical facts, but not as to conclusions of law.

"They may also be examined as to conclusions of science drawn from particular experiments. But not as to their opinions on matters not of their particular science.

"Examinations made *ex parte*, when there could have been notice to the opposite side, are inadmissible. But investigations by persons having intimate and continuous opportunities of examination are not excluded because *ex parte*, when these investigations would not be enhanced in accuracy and authoritativeness by being preceded by notice to the opposite side." (Wharton & Stille, §§ 1243—1247.

CONCLUSION.

It will be seen from the foregoing citations, that what with the adverse rulings of the courts as to what questions may and what may not be answered by the expert; with the case complicated by questions upon hypothetical statements of fact, so framed as to bring out the answer most favorable to the side propounding them; with the notable difference or seeming difference of opinion among medical witnesses themselves; that the "expert's" position is not a very enviable one, and that there is an urgent necessity for so modifying the laws bearing upon this kind of testimony, that the "*best*" may be had in every case.

To the plans proposed by Professor Washburn and others, already cited, I do not know how I can serve the profession better than by transcribing in full § 1250 of Wharton & Stille's Medical Jurisprudence, bearing upon the same subject.

In the medico-legal system in force in Germany, there is first a county or city physician, who has been scientifically trained in medicine, surgery and obstetrics. In addition to this, he is to prepare himself and be examined in the specialty of medical jurisprudence, which examination is to be conducted by the supreme medical board of the State. Associated with the city or county physician, there is a city or county surgeon. These may differ in opinion, in which case there is an appeal taken to the medical tribunal of the province (State); finally, an appellate court medical for the whole monarchy. Messrs. Wharton & Stille, after developing the above principles, go on to state, § 1250 :

"A similar system could be readily grafted on our American practice. We are familiar with army physicians and army surgeons, and of subordination in rank in these officers. There would be no difficulty in providing in each county for a county physician, who by tests of an adequate competitive examination, shall show his general and special competency for this particular post. In addition to the duties devolved on him of conducting post mortem examinations, and of pursuing any other investigations that may be required in a litigated issue, such a physician might be made the arbiter in those moot questions by which the law has been kept in a state of such distressing incertitude. Is there such a disease as moral insanity or as *mania transitoria?* Can human blood stains be distinguished after having become dried? If a question of this kind arise on the trial of a cause, it would not be inconsistent with the analogies of the law to refer it to an official expert, just in the same way that a chancellor sends a question of fact to be determined by a master in chancery or by a common law court and jury. But if this be done, it should be done with the checks that attend the chancery system which has just been noticed. The official physician who acts as referee must be placed under judicial restraints. He should owe his appointment to neither party, but to the

State, irrespective of any particular case. His duty it should be to take testimony, if needed, on the case, and to hear counsel, so that he will be in no danger of hazarding one of those rash and ignorant opinions which have so much disgraced this branch of medical practice. After thus judicially hearing the case, it should be his further duty to judicially certify his opinion to the court by whom the reference is made. In proper cases there might be allowed an appeal from such opinions to a supreme court of governmental experts appointed by the State at large.

"It may be said that this may be productive of occasional delay. This is true; but the difficulties thus arising would not be so great as those which almost every contested medical issue now involves, and which in cases of insanity, have led courts so often to grant new trials from sheer despair of drawing a decisive conclusion from the jargon thus introduced. Soon, also, the delay of appeals would be reduced; for certain great cardinal questions would be settled beyond dispute. We should soon know whether there is such a thing as moral insanity, and whether it is practical to distinguish human blood after the expiration of a week from the period of its drying. Settle a few of such points as these, and we relieve criminal justice of a large part of the uncertainties by which it is now beset, and we will have a series of rules, by which cases can be intelligently, consistently and humanely conducted. Nor will this be all. We will be able to get the judicial utterances of science as to vexed issues of fact, instead of the interested arguments of experts, who are virtually employed as counsel by the party calling them, or the wild utterances of philosophic monomaniacs who are called simply because of their absorption in some unique theory of their special concoction. Such men need not be silenced. Experts as counsel, indeed, will find a proper and important office in presenting the two sides of the issue to the expert who acts as referee. But the expert who fills this last judicial post will be disembarrassed of all personal relations. He will have no client to serve and no past partizan extravagances to vindicate. He will render his opinion as the advocate neither of another nor of himself. When he speaks, he will do so judicially, as the representative of the sense of the special branch of science which the case invokes, governed by the opinion of the great body of scientists in this relation, and advised of the most recent investigations. When this is done, we will have expert evidence rescued from the disrepute into which it has fallen, and invested with its true rights as the expression of the particular branch of science for which it speaks."

A last rule for the government of the medical expert is this: Do not be too free in the expression of opinions off the witness stand. Time and again physicians are subpœnaed as witnesses, and their knowledge used by counsel in the management of the case, after which they are not called upon the stand and receive no fee for their attendance beyond the ordinary witness fee. Require for such opinions the same remuneration you would upon the stand. Give no opinion till such fee is paid.

The above is deduced from some personal experience which it may not be out of place to relate in this connection.

In the case of the *People* v. *Swanson*, alleged murder by poisoning, taken by change of venue from Knox to Warren Co., State of Illinois, the attorneys for defendant availed themselves of my library in preparing themselves for the trial. My knowledge of those matters was also solicited, under an express agreement that I would not be subpœnaed in the case. Under such conditions I rendered what aid I could, as the attorney who availed himself of my courtesy was a personal friend. My surprise was very great, at a visit from the sheriff, just before the sitting of the court, with a notice to appear and testify as to what I knew in the case. This invitation I did not feel at liberty to refuse. During the progress of the case both myself and the chemical expert for the defense were consulted frequently in respect to certain testimony given for the people, and when it was learned that we both coincided with the opinions of these witnesses, I was excused from further attendance, and I believe also the chemical expert. For the attendance of the better part of two weeks, a witness fee of some ten or twelve dollars was subsequently paid me by the people.

BIBLIOGRAPHY.

*American Law Review*, 1866—1871.
GREENLEAF, On Evidence.
STARKIE, On Evidence.
REDFIELD, On Wills.
ORDRONAUX, Jurisprudence of Medicine. 1869.
TAYLOR, Principles and Practice of Medical Jurisprudence.
TAYLOR, Medical Jurisprudence. Philadelphia. 1866.
ALLISON'S Practice.
RENOUARD'S History of Medicine.
ROSCOE, On Criminal Evidence.
BLANFORD, Insanity and its Treatment. 1871.
RAY, Contributions to Mental Pathology. 1873.
RAY, Jurisprudence of Insanity. 1871.
ELWELL, Malpractice and Medical Evidence. 1866.
BECK, Medical Jurisprudence. 1863.
WHARTON & STILLE, Medical Jurisprudence. 1873.
PHILLIPS, Circumstantial Evidence. 1873.
GUY, Forensic Medicine. London. 1868.
CASPAR, Forensic Medicine. *New Sydenham Soc.*
PEUGNET, Gunshot Wounds of Abdomen.
*Medical Record*, New York. 1866—1874.
*Medical Times*, Philadelphia. 1870—1874.
DUNGLISON, Medical Dictionary. 1866.
*Medical and Surgical Journal*, Boston. 1850.

